# Impact of extreme weather events and climate change for health and social care systems

**DOI:** 10.1186/s12940-017-0324-3

**Published:** 2017-12-05

**Authors:** Sarah Curtis, Alistair Fair, Jonathan Wistow, Dimitri V. Val, Katie Oven

**Affiliations:** 1 Department of Geography, Durham University, Durham, DH1 3LE UK; 2 Edinburgh School of Architecture & Landscape Architecture, University of Edinburgh, Edinburgh, UK; 3 School of Applied Social Science, Durham University, Durham, UK; 4 School of Energy, Geoscience, Infrastructure and Society, Hariot-Watt University, Edinburgh, UK

## Abstract

This review, commissioned by the Research Councils UK Living With Environmental Change (LWEC) programme, concerns research on the impacts on health and social care systems in the United Kingdom of extreme weather events, under conditions of climate change. Extreme weather events considered include heatwaves, coldwaves and flooding. Using a structured review method, we consider evidence regarding the currently observed and anticipated future impacts of extreme weather on health and social care systems and the potential of preparedness and adaptation measures that may enhance resilience. We highlight a number of general conclusions which are likely to be of international relevance, although the review focussed on the situation in the UK. Extreme weather events impact the operation of health services through the effects on built, social and institutional infrastructures which support health and health care, and also because of changes in service demand as extreme weather impacts on human health. Strategic planning for extreme weather and impacts on the care system should be sensitive to within country variations. Adaptation will require changes to built infrastructure systems (including transport and utilities as well as individual care facilities) and also to institutional and social infrastructure supporting the health care system. Care sector organisations, communities and individuals need to adapt their practices to improve resilience of health and health care to extreme weather. Preparedness and emergency response strategies call for action extending beyond the emergency response services, to include health and social care providers more generally.

**Electronic supplementary material:**

The online version of this article (10.1186/s12940-017-0324-3) contains supplementary material, which is available to authorized users.

## Background: Conceptual framework and purpose of this review

This review, commissioned by the Research Councils UK *Living With Environmental Change* (LWEC) programme, concerns research on the impacts on health and social care systems in the United Kingdom (UK) of extreme weather events under conditions of climate change.

We conceptualise the health system following the World Health Organisation (WHO) definition:

*A health system is the sum total of all the organizations, institutions and resources whose primary purpose is to improve health. A health system needs staff, funds, information, supplies, transport, communications and overall guidance and direction. And it needs to provide services that are responsive and financially fair, while treating people decently* [1].

We extend this definition to cover health and social care and, as proposed by other authors [2–4], we consider *physical*, *institutional* and *social* infrastructures as part of a complex care system. Physical elements include ***built infrastructures*** (not only individual buildings but also networks of health care facilities and utilities and transport networks). ***Institutional infrastructures*** are comprised of institutional policies and practices, human resources and professional practice***. Social infrastructures*** include community groups operating in the informal sector and individual’s social networks and practices.

We aimed to assess the main policy relevant messages from research relevant to care systems the UK. Extreme weather of relevance here includes: events involving temperatures which, in the UK context, constitute extremely hot or cold weather; prolonged or intense precipitation causing flooding.

We have framed this review to assess two sets of questions: first, what is the evidence concerning *observed* impact of these events on service operability and access and on pressure of service demand on the care system; second, what is the evidence regarding potential *future* impacts of extreme weather on health services in light of projected climate change (as projected up to 2050–80) and potential for adaptation of health and social care infrastructure in the context of climate change. This review focuses on evidence relevant to the UK, however we discuss in the concluding section how our approach and findings may also relate to health and social care systems internationally.

## Review method

We chose not to attempt a fully systematic review since some formal systematic review procedures [5] are inappropriate for the type of problem and application of evidence considered here. We also considered it inappropriate to attempt meta-analysis or statistical or probabilistic assessments of the research evidence because: few studies that are relevant to this review follow strictly comparable methodologies; measures of change being used are quite diverse; some useful studies are qualitative not quantitative; the scope and scale of focus for the research reviewed is variable (including research at national, regional and local scales and various outcomes). We therefore adopted a ‘structured’ review approach, adapting the procedures required for formal systematic reviews.

The aims of the review were specified as shown above. We employed search and selection criteria designed to select research related to impacts of extreme events on factors relevant to health care system functioning, for which we were able to assess the rigor and quality of the research using quality criteria relevant to the type of (quantitative or qualitative) method used. We also selected papers reporting research related to the kinds of events and infrastructure relevant to health care functioning in the UK. We have not included papers reporting original research and reviews focussed on other country contexts, since, although there may be ‘parallel’ experiences in other countries, it is important to concentrate on the climate risks and built, organizational and social infrastructure in the UK national context [6]. Some more international studies were included if they reported on internationally comparative work including the UK, or proposed theoretical frameworks which seemed to have relevance to the UK setting.

The review procedure involved a first stage using an ‘umbrella review’ approach [5], drawing upon literature reviews previously prepared for projects supported by the LWEC *Adaptation and Resilience to Climate Change* programme (http://www.nerc.ac.uk/research/partnerships/ride/lwec/) and by ESRC and EUFP7 (see acknowledgements). A number of key reviews and assessments produced recently for government agencies were also considered [7–17]. In the second stage these reviews were supplemented by a ‘structured’ automated search for relevant literature published 2010 – June 2017, on the online search engines *Web of Science* and *PubMed,* using the following terms: ‘climate change’, ‘heat’, ‘cold’, ‘flood*’ in combination with ‘health care’, ‘health services’. From the results of the automated search we made a manual selection of relevant articles meeting our criteria.

## Review findings regarding observed impacts of extreme weather on health service infrastructure

In this section we summarise evidence regarding the observed impacts of extreme weather on health and social care systems in the UK under contemporary climatic conditions.

### Impacts of heatwaves

Heatwaves may impair functionality of hospitals including medical equipment and storage of medicines [8, 18, 19], and affect thermal comfort of hospital buildings for patients and staff [20–22]. *Structural design to meet other requirements in a hospital may compromise thermal regulation,* e.g. due to poor heating system control strategies, or safety protocols restricting window opening [23–25, 21, 26, 27]. Modern or temporary buildings may perform worse in terms of thermal regulation than older buildings [22, 28, 21, 29]. There is relatively little evidence on impacts of extreme heat in other facilities, such as care homes, though some case studies suggest problems associated with poorly adapted equipment, structural design and care practices [2, 30], and lack of awareness among designers and managers regarding the need for heat management as well as avoiding risks of cold [31, 32]. More research is needed on the extent of such problems, especially since mortality risks during heatwaves are particularly high for older people in care homes [33–36]. Impact of extreme heat on the wider networks of built infrastructure and utilities supporting health care systems are generally insignificant given the levels of extreme temperatures observed to date in the UK [37–39].

Health care activity impacts of heatwaves in the UK depend on the health service response to changes in population health. There is regional variation in the absolute temperatures treated as critical for heat alert intervention under National Health Service (NHS) protocols [40], triggering action including additional checks on vulnerable patients. Hospital admissions for respiratory diseases increase during heatwaves [41]. During very hot weather syndromic surveillance data show increases in GP activity [42] and emergency department use for some conditions likely to be associated with heatwaves, and in ambulance call-out rates [43–45] as well as additonal demand due to less urgent events such as arthropod bites [46]. Heatwaves also have adverse consequences for patients being treated for other conditions including cardiovascular diseases and mental illness, though these seem not to give rise to additional demands for health care [47–50].

### Impacts of coldwaves

Utility systems such as power supplies are unlikely to be affected during coldest conditions observed in the UK [37, 38]. Other infrastructure, particularly transport systems, are likely to be disrupted by snow and ice, creating difficulties of access for patients needing to use health facilities and for domiciliary care staff in reaching their clients in their homes [37, 51–54]. Ambulance response times also fall during very cold weather [45].

Impacts on health service activity arising from cold waves include increased rates of consultation and treatment for respiratory conditions and emergency treatment for injuries resulting from falls in ice and snow and the risks, especially among older people [55–57]. However, hospital admissions due to cardiovascular diseases have not been observed to increase due to coldwaves, although cardiovascular mortality rates do increase [58]. Individual physiological risk factors such as age and pre-existing health problems have been shown to relate to risk of health impacts from coldwaves and these may also affect variation in use of health services. Statistical evidence for socio-economic risk factors affecting coldwave impacts is not robust [59–61].

### Flooding impacts

Flood risks are locally variable, depending on flood risk zones and flood return periods [35]. Evidence from European countries including the UK [14] suggests that ***impacts on physical infrastructures supporting health services*** include flooded health facilities (sometimes resulting in evacuation of patients), interruptions to power and water supplies, safety of and access to patient records, interruption to ambulance services, continuity of outreach and community care. Wider infrastructure systems may have a degree of tolerance of local flooding. For example electricity substations in the UK should be resilient to flooding up to a depth of 300 mm [37, 38]. However, flooding to less than this depth may interrupt service access and delivery [14, 37, 51, 52, 54], and areas that are at greatest risk in some parts of the UK have populations that are particularly likely to need health services [37, 62].

Direct physical health impacts of floods in the UK include relatively rare risks of: drowning; injury by submerged or floating debris, fire or electrocution; toxicity or infection linked to water shortages or contamination, heart attacks [9, 14]. There is also some evidence of mental and physical morbidity associated with exposure to flooding with potentially more long term and significant impacts on the health care system [14, 63, 15, 35, 64–68]. It is recognised [14] that research is limited on how use of health services is impacted by flooding, although analysis using data from Syndromic Surveillance systems is beginning to address this question [69].

## Findings regarding potential future impacts of extreme weather on health services in light of projected climate change and potential for adaptation

Research estimating future impacts of extreme weather events in the UK on health service demand faces challenges including: relative imprecision in projections for extreme weather conditions; limited consideration of local factors such as urban heat island effects; difficulties in anticipating levels of adaptation to extreme events [70–72]. All climate projection models depend on assumptions about the future trends in factors driving climate change, including the ‘emission scenarios’.

Forecasts for climate change in the UK suggest more frequent heat waves [73] and (with less certainty) increasing fluvial and pluvial flooding due to intense rainfall and river and surface runoff [74–77]. Higher sea levels are predicted, with more frequent coastal flooding [11, 78]. Cold wave risks are likely to reduce long term, but may continue in the shorter term, due to sea ice influences [79]. The *speed of change* in risk of climate impacts, as well as the *level* of risk, may be important for future capacity of local health services to cope with extreme weather. Areas of the country where the *change* in risk of heatwaves may be greatest up to 2030 are not necessarily those where the *probability* of heatwaves will be greatest [37].

Reviews of climate change risk assessment for the UK health sector [7, 12] suggest future trends in hospital activity associated with extreme weather and associated with population growth and aging, and predict increases in hospitalisation rates and rising costs of hospitalisations to 2030 due to increased ozone concentrations and heatwave events. However, it is acknowledged that these predictions are not certain, and methods do not account for local heat island effects in major cities, or possible future health system adaptations which might alter medical care models and service use during extreme weather events. For example, given the development of real time syndromic surveillance systems we might speculate whether health services might in future aim to reduce emergency call outs and hospital or care home admissions, for example by proactively mitigating excess heat related- cardiovascular mortality among the populations most at risk or expanding online health advice systems. Also we have relatively little information on how primary and ambulatory care services may be impacted by extreme weather events in future. Attempts to model the cost effectiveness of implementing strategies such as cold weather plans for the NHS are limited by lack of detailed information on the extent to which these plans are being implemented and targetted towards those most in need [80].

International research findings from other countries may give some indication of conditions that might in future apply to the UK and we focussed particularly on those which included comparison with the UK. Research comparing changes in hospitalisation rates associated with maximum apparent temperature in 12 European Cities including London [41] showed that increases in admissions during heatwaves were more pronounced in warmer Mediterranean cities than in Central and Northern European cities, perhaps because in Mediterranean regions heatwaves involve more extreme temperatures presenting the greatest physiological risks for health. These conditions in Southern Europe might be indicative of future patterns in the UK as our climate changes. However, such simplistic international comparisons may not be reliably predictive of future conditions in the UK, since they do not reflect other contextual differences among countries, such as living conditions, behavioural adaptation to extreme weather and health service organisation.

International reviews of flood risks for health [17, 81–85, 14] show that the reported scale of current impacts observed internationally is quite variable. We did not identify any original research on health system impacts of floods using rigorously internationally comparative data and including the UK. We know little about the longer term health impacts of floods in the UK, which may affect patterns of service use [86]. International findings show significant but variable (8.6%–53%) increases in prevalence of psychiatric morbidity [86], and other reviews conclude that post-traumatic impacts of flooding are significant [87, 88]; so these may result in increased demand for health services.

Therefore, evidence to inform adaptation of health and care systems to future impacts of extreme weather under conditions of climate change in the UK remains rather limited, and this needs to be borne in mind when considering potential adaptive strategies.

## Potential for adaptation of infrastructure design and practice for improved preparedness and performance during extreme weather

A number of sources argue that in addition to mitigation of climate change through more environmentally sustainable practices in care systems [89–91], strategies to adapt to climate change are important. Commentators recommend the establishment of national health adaptation plans and action involving partners in the health and social care system generally, not only the emergency response services [92–98]. Adaptive strategies should address considerations including: long term protection by ‘climate proofing of settlements, institutions and societies’, equity and difference in vulnerability and cost effectiveness [35]. Below we summarise relevant strategies for adaptation of built infrastructure and social and institutional infrastructures, within a ‘whole system’ perspective.

A number of changes have been proposed for the UK, many of which relate to ***design of individual buildings*** rather than whole infrastructure systems. With respect to adaptation to more frequent heatwaves, technical guidance from the Government may be referenced, particularly when new hospitals are constructed [23–25]. However, increased use of mechanical cooling would undermine the NHS Carbon Reduction Strategy and contravene the Climate Change Act [21, 22]. Rather than referencing crude thermal comfort maxima for building design (CIBSE) (ASHRAE) concepts of adaptive older incomfort, referencing temperatures recently experienced might be more appropriate (BS EN 15251) [99].

More sustainable solutions [100] including attention to building mass and orientation may be used to positive effect, as has been done in the past [29]. Refurbishment of existing hospital buildings could improve thermal comfort [21, 22] and solar shading options may help to reduce internal temperatures. A low-energy new-build hospital [100] features: a building of 3–4 storeys, with courtyards facilitating ventilation strategy; air ducted through the building after being cooled naturally in a low level chamber; ‘thick’ walls; openings shaped and sized to reduce undesirable solar gain whilst preserving daylighting; single patient rooms naturally ventilated in ways other than opening windows. Options for low-energy refurbishment of existing, 1960s hospital buildings could halve their energy use [22]. Advanced Natural Ventilation can be devised to move air through a building to achieve even greater energy efficiency [100–102]. Similar options for 1970s courtyard hospital designs have also been presented [28] and refurbishment of pre-1948 buildings should aim to further improve further the higher resilience of this type of structure, whilst enhancing patient privacy and dignity with design features such as individual patient rooms. These options can be executed in stages and are likely to achieve long term savings through greater energy efficiency. They might need to be applied variably across the country, given regional differences in climate, and the measures proposed could provide effective solutions up to the 2040s [103].

For the ***wider built infrastructure system***, adaptation to increasing risks posed by flooding is a priority [14] as well as measures to ensure continued resilience to cold weather events. A ‘whole system’ approach taking into account interdependencies between different infrastructure systems, is required and local level network flow models can assist the development of cost effective adaptation strategies [104, 105]. The ARCC BIOPICCC project also pioneered the use of the NHS Strategic Health Asset Planning and Evaluation (SHAPE) toolkit [35] as a way to identify parts of the infrastructure for which adaptation may be most needed and this toolkit now includes flood risk information [106]. More specific adaptation strategies include modifications to equipment, such as the use of winter tyres for ambulances during cold weather [45], installation of emergency generators at key health facilities to ensure continuity in power supplies [104], or strategic planning for future shortages of essential supplies [107].

Action for ***adaptation of social infrastructure*** should include enhanced understanding of *individual* vulnerability and action to modify individual physiological, psychological and behavioural factors, and measures to modify collective *social and institutional* factors.

Internationally comparative studies of ***individual adaptation*** [108, 109] suggest protective behaviours to improve resilience to cold, such as clothing and central heating, are more common in colder regions. Changes in such behaviours over time in the UK may have contributed to reductions in cold weather mortality in London [110]. Case study research [111] shows some individuals take what they consider effective health protective measures during extreme weather events. However, there is little research on whether such behaviour is widespread among those most at risk in the UK population, and it is recognised that public information campaigns may have limited effect [7]. One UK study [36] cited in an international review [112] showed participants were unaware they were at particular risk due to hot weather.

Few studies have assessed whether public awareness campaigns are effective for the most socially isolated and marginalised groups, who may be most vulnerable, but there is evidence that many people do not practice the public health advice they receive, e.g. older individuals do not always consider themselves vulnerable [106, 113]. Extreme weather warnings can be confusing when issued from different sources and appearing to offer differing advice [112]. It is difficult to assess whether public awareness is improving over time, since many information programmes have only recently begun, extreme weather events tend to be rare, and the same populations may not be exposed repeatedly to similar events [112].

***Institutional and collective adaptation*** for resilience to extreme weather includes enhanced risk governance and professional practice, and action in wider society to change collective social processes [92]. Several authors call for *improvements in evaluation of, and response to risks*. An adaptive capacity index has been proposed, representing potential for institutional adaptation in terms of *risk identification, risk reduction, disaster response and adaptive governance* [2], and related research revealed that adaptive capacity was limited in most of the social care institutions surveyed, highlighting that management of heatwave related health risks for older people tends to be seen as an issue for emergency services rather than institutions such as care homes [114]. There are calls for greater advocacy in the health system regarding climate change issues [115] and for medical education to cover climate change risks so that medical staff understand the issues [116] and are better placed to act. Most disaster response mechanisms operated through normal, routine health care checks, rather than special procedures for extreme weather. Research is beginning to evaluate early warning systems that may enhance preparedness for extreme weather [117].

Enhanced ***risk identification*** involves *understanding* the factors making certain groups in the population relatively vulnerable, *recording* information on those most at risk, and *acting* on the information to target relief efforts towards these groups during extreme weather events. Local indicators of potential vulnerability to climate change have been compiled for localities in England [4, 118, 119]. Risk registers allowing community groups and professional carers to locate individual vulnerable people are important, but increasingly difficult to maintain as service provision becomes fragmented among various agencies in the UK, raising questions about data sharing, confidentiality and compatibility [113, 120]. Data from real time surveillance systems may become important to assess changes in the health and social care system, issuing alerts prompting more effective response to extreme conditions [44, 57].

The international literature argues for ***more integrated planning and preparedness*** for extreme weather, acknowledging the complex interdependencies involved [121], and the Sendai Framework for Disaster Risk Reduction underlines in an international context why this is important for health and wellbeing [122]. Commentators have underlined the need for interdisciplinary holistic strategies [123] to integrate robust, consistent scientific knowledge with local knowledge to improve resilience [70, 124], to emphasise sustainability and sustainable development [125] as a concern for health and social care systems, and to integrate human health and environmental impact analysis more effectively [126]. A growing emphasis on complexity theory and ‘nexus’ thinking [106, 127] provides frameworks that may help agencies and communities to develop strategies for preparedness for extreme weather that consider the networks of built, institutional and social infrastructures that are important for resilience.

Local government policies in place are not always effectively implemented on the ground [52, 54, 65, 128]. Sustainable care should be based on interdisciplinary research, co-production, mutualism and localism [129, 130] and local knowledge needs to be integrated more effectively with environmental science on risks such as flooding [4, 131]. Research reports effective approaches using local case studies [54, 132, 133, 106, 134]. Similar emphasis on integrated planning comes from the international literature [71]. In Canada, Critical Systems Heuristics (CSH) was helpful in engaging stakeholders, and addressing issues of power relations between collaborating partners [135] and recent research has compared and reviewed reports of a variety of toolkits now available [136]. International literature also emphasises stakeholder engagement and locally adapted frameworks to access critical social infrastructure involving connectedness, collaboration and adaptive response. While our review was not internationally comprehensive, we note examples of research in Canada [131, 137], the USA [138, 139], the UK [120] and Australia [140], with calls for coordinated action to: reduce heat exposure; improve access to cooling; adapt the built environment; enhance surveillance and early warning systems and public awareness communications [141]. Reviews also emphasise the need for a greater attention to ***equity and inclusivity***, drawing attention to the inequalities between socio-demographic groups in exposures to and impacts of extreme weather [142] and the increased care burdens that women are expected to undertake during disasters involving extreme weather events, that may be unsustainable [132]. Collective action at local level must pay attention to inclusive representation of subgroups within communities [143].

## Conclusion

This review has highlighted a number of general conclusions which are likely to be of international relevance, although here we have focussed on the situation in the UK. Extreme weather events impact the operation of health services through the effects on built, social and institutional infrastructures which support health and health care, and also because of changes in service demand as extreme weather impacts on human health.

It is challenging to accurately forecast the likely pattern of extreme weather events in future decades, especially for local areas within the UK. However, it seems likely that, particularly for heatwaves and floods, the frequency will increase and the pattern will be regionally variable. Strategic planning for extreme weather and impacts on the care system should be sensitive to within country variations. The impact of these changes on population health and health care systems will depend in part on adaptation to these changes. More research is needed to examine the implications of different scenarios for adaptation as well as for climate change.

The recent literature reports on increasing use of new methods of tracking and studying impacts of extreme weather on health systems, including syndromic surveillance systems in the UK. We have noted arguments in the literature that it may be necessary to find ways to incorporate more qualitative ethnographic information describing individual experiences, or perhaps to be more responsive to lay reports. The growth in use of sources such as social media to track impacts of extreme events in real time provides one mechanism for this, which we may expect to see extended in countries like the UK.

Adaptation will require changes to built infrastructure systems, not only by making individual buildings, such as hospitals, more resilient, but also by action to modify the whole network of infrastructure supporting the health care system, so research on the impacts of extreme weather and resilience of utilities, transport and communications systems needs to be brought to bear in adaptation of health services to climate change.

Institutions and communities need to adapt their practices to improve resilience of health and health care to extreme weather. Preparedness and emergency response strategies call for action extending beyond the emergency response services, to include health and social care providers more generally. Adaptation and preparedness measures may be hampered by pressures of wider trends, expressed in terms of reduced expenditure and competing demands on health care systems like that in the UK, which are mainly publicly funded. We have noted above some examples of econometric studies which have begun to explore the cost effectiveness of measures to make health and social care more resilient to extreme weather.

More research is needed to assess current and future impacts of extreme weather on primary and ambulatory services and how effectively national governmental advice is filtering through the health and social care system to affect practice on the ground, and to evaluate the various sources of guidance and ‘toolkits’ now available. Communities have an important role to play in adaptation and resilience, and research is only beginning to accumulate on how far changing behaviour and practices by individuals and communities, as well as professional carers may modify the potential impact of extreme events under conditions of climate change.

## Open peer review

Peer review reports for this article are available in Additional file 1.

## Additional file

Additional file 1: Open peer review (PDF 191 kb)
