# Supplementary material for: Impact of extreme weather events and climate change for health and social care systems
Source: Environ Health. 2017 Dec 5;16(Suppl 1):128. doi: 10.1186/s12940-017-0324-3 (PMC5773887; doi:10.1186/s12940-017-0324-3)
Supplement: Additional file 1: — Open peer review (PDF 191 kb) [file 12940_2017_324_MOESM1_ESM.pdf]

Reviewer reports

Title: Impact of extreme weather events and climate change for health and social care systems

Reviewer 1: Sabrina McCormick

This article is a review of the literature regarding locally variable impacts of extreme weather events on health care systems in the UK, both in the present and this century as a result of a changing climate.

The first line of the introduction is difficult to understand, but seems to suggests that the authors will review a report. The background section then articulates that the paper will review the literature. This should be corrected. Early text written in bullets and should, instead be written in narrative form.

A literature review should not be ad hoc, but systematic. An ad hoc review is not an effective methodology. This lack of systematization is reflected in the first section on heat waves and infrastructure. In that section, the authors review some literature on the health effects of heat waves and attempt to extrapolate from those findings to how health care systems could be affected. This might be a fine activity for a discussion section, but not the primary body of the paper. This approach is a problem, in part, because the review of health effects is so ad hoc and slim. The first paragraph of that section about infrastructure should consider energy systems and the grid, more generally, as well as infrastructural factors that affect caring for those in-place when an extreme event occurs. Much of the healthcare necessary during such events will not happen in a hospital context. Power is considered in the flood section and should also be considered in the heat section.

I suggest that the section "Findings regarding potential future impacts of extreme weather on health services demand due to mortality and morbidity in the UK" should come first before the more focused sections on specific kinds of events as a way to focus the paper.

There are certain statements that need much greater substantiation. For example: "A number of sources argue that in addition to mitigation of climate change through more environmentally sustainable practices [83-85], strategies to adapt to climate change are important." This sentence does not sufficiently summarize an extremely large literature on adaptation. This is true of several other sentences in this paragraph.

Another example is: "Few studies have assessed whether public awareness campaigns are effective for the most socially isolated and marginalized groups, who may be most vulnerable, but there is evidence that many people do not practice the

public health advice they receive, e.g. older individuals do not always consider themselves vulnerable." That can be referenced with a number of sources.

The most interesting part of the article starts in paragraph three of the section on improving infrastructure design. Here, it seems like the authors have thoroughly reviewed the literature. However, when the paper gets to the individual-level factors, it is very superficial and misses literature that has been produced in other countries but that could be applied to the UK context.

It seems that the section on risk identification is also referring to vulnerability, but does not reference the large literature that discusses related factors.

Overall, this piece should be clearer and more limited about its scope by clearly stating up front the extreme weather event risks and the effects thereof they intend to address. Right now, the scope is so broad, it's almost impossible for them to execute the review effectively. That is additionally difficult without a more rigorous review method. I suggest that they also develop a standard approach to each section in order that the review is more digestable.

Declaration of competing interests: I declare that I have no competing interests.

Reviewer 2: Linda Varangu

Thanks to the authors for tackling this topic as it is of high importance for the health care sector around the world. In Canada we follow with great interest the leadership demonstrated by the UK and NHS on climate change resiliency and adaptation in the health care sector.

The paper as written is recommended for publication. A few comments with respect to the reported results are provided.

The broad objective of this research was to determine what information from peer-reviewed journals were available to help inform the effects of climate change, in particular extreme weather events of heatwaves, coldwaves and flooding, on the health and social services sector in the UK.

What this paper has shown is that according to the information presented in the peer-reviewed publications, there is still much to learn and to research when it comes to solid reporting on the impacts of climate change on the health care sector. While this is very important to know, it would also help if the authors could help us also understood some of the surrounding issues related to this observation and see how more of this reporting can be encouraged, especially in a more timely manner. We see in Canada, for example, some increase in reporting in the lay literature on extreme weather impacts on the health care sector particularly over the past several years, but this reporting is generally not accompanied by rigorous academic evaluations, nor is much of it get reported in peer-reviewed journals.

The authors may want to consider the following observations on the challenges the health care sector representatives have in reporting on the impacts of climate change, which can include the following:

- Reporting by the health care sector on the impacts of climate change events and the effects on their operations and facilities, is generally targeting funders and community stakeholders.
- While we see some new reporting in the lay literature of the increased impacts of extreme weather, detailed assessments on these impacts on the health care sector are not easily available even in the lay literature. We know from media reports that impacts have occurred, such as temporary hospital closures due to power failures during extreme weather, but follow-up information is not easily available. This may be due to numerous competing health care sector priorities, lack of staff to undertake these more detailed reports, and engagement of staff who may not be well informed on the relationship of these events with the larger realm of climate change impacts.
- Situation analysis from impacts of extreme weather on the health care sector has generally tended to deal with lowest cost, short-term fixes.

- Reflections on the longer-term system changes that would elaborate on the potential of preparedness and adaptation measures that may enhance resilience are not common, as the health care sector has generally not taken this long-term assessment perspective. For primarily publically funded health care (which both the UK and Canadian health systems are), planning in the health care sector is associated with funding, and that is tied to the funding decision, and regulatory requirements set by the parties in power.
- Some health care sector members may be concerned that reporting on the effects of extreme weather on their facilities may be perceived as poor operations on their part, or that their facilities have not responded in ways that would attract future funders to their foundations. In such cases public reports would not be easily available.

In order to plan for appropriate adaptation responses for the health care sector, we will need more information from the health care sector on what their impacts have been, and to have this information in a timely fashion, and assessed in a rigorous manner. Reporting could be facilitated by providing guidance to the health care sector on what information to report, such as through a standard reporting format, that will allow the further analysis by the academic community. This would require a greater partnership (and funding) for these types of analysis but could stimulate more publications in peer-reviewed journals, or more detailed reports in the lay literature that may be acceptable for research reviews such as this one.

Competing interests statement: I declare that I have no competing interests

Author response

Authors: We'd like to thank the editor and two reviewers for their comments on this paper. A revised version is attached showing the edits made in tracking. Below are the points raised by the editor and the reviewers with a note of our response to these.

Supplement Editor's comments (Kristie L. Ebi)

The reviewers identify several areas where additional efforts are needed to improve the quality and clarity of the manuscript, , better referencing of statements, putting the research into context of other issues, and other issues discussed in the reviews.

I suggest elaborating on your approach (including criteria) to conducting the structured review and clarifying the reasons for not conducting a systematic review.

Response: While trying to keep close to the word limit for this journal we have made the following changes (as detailed in the responses below to the reviewers who raised helpful suggestions):

- **Clarified our structured review approach (if required because of the word limit, we could move this more detailed explanation to an electronic appendix, but we have included it in the main text to respond to the reviewer who raised queries about it);**
- **Extended the review using the same structured review approach to include references published since July 2015, when this paper was originally submitted;**
- **Clarified some of the arguments regarding the aims and objectives and the interpretation;**
- **Included reference to the wider international issues for which this review is pertinent (while explaining that given the purpose to inform national policy, this review has concentrated on research relevant to the UK).**

Below we have copied the reviewers' comments and indicated our response.

Reviewer 1 (McCormick)
Reviewer: This article is a review of the literature regarding locally variable impacts of extreme weather events on health care systems in the UK, both in the present and this century as a result of a changing climate. The first line of the introduction is difficult to understand, but seems to suggests that the authors will review a report. The background section then articulates that the paper will review the literature. This should be corrected. Early text written in bullets and should, instead be written in narrative form.
Response: This is helpful feedback. We have rewritten the introduction to the paper. In particular, we now state in the introduction that:
This review, commissioned by Research Councils UK Living With Environmental Change (LWEC) programme, concerns research on the impacts on health and social care systems in the United Kingdom (UK) of extreme weather events under conditions of climate change.

Then we explain the aims of the review in cursive text, to replace and clarify the previous explanation that was previously summarised in bullet points.
We aimed to assess the main policy relevant messages from research relevant to care systems the UK. Extreme weather of relevance here includes: events involving temperatures which, in the UK context constitute extremely hot or cold weather; prolonged or intense precipitation causing flooding.
We have framed this review to assess two sets of question: first, what is the evidence concerning observed impact of these events on service operability and access and on pressure of service demand on the care system; second, what is the evidence regarding potential future impacts of extreme weather on health services in light of projected climate change (as projected up to 2050-80) and potential for adaptation of health and social care infrastructure in the context of climate change. This review focuses on evidence relevant to the UK, however we discuss in the concluding section how our approach and findings may also relate to health and social care systems internationally.

Reviewer: A literature review should not be ad hoc, but systematic. An ad hoc review is not an effective methodology. This lack of systematization is reflected in the first section on heat waves and infrastructure. In that section, the authors review some literature on the health effects of heat waves and attempt to extrapolate from those findings to how health care systems could be affected. This might be a fine activity for a discussion section, but not the primary body of the paper.
Overall, this piece should be clearer and more limited about its scope by clearly stating up front the extreme weather event risks and the effects thereof they intend to address. Right now, the scope is so broad, it's almost impossible for them to execute the review effectively. That is additionally difficult without a more rigorous review method. I suggest that they also develop a standard approach to each section in order that the review is more digestable.

Response: We certainly respect this reviewer's argument that fully systematic reviews are considered, especially in fields such as medicine, to be superior to other methods. However, we have revised the explanation to expand on our position, that a fully systematic review procedure would not be feasible for the kinds of evidence which need to be reviewed here, and that we have, nevertheless, applied a 'structured' review approach with a specified search procedure and clear criteria for inclusion. The method we have used has proved acceptable for other well respected peer reviewed journals. The word limit does not allow us much space to elaborate on what we agree is an interesting debate on how to design robust and valid review procedures suitable for the kinds of broad policy issue addressed here. However, we have rewritten the description of our approach, in the section entitled 'review method', as follows, to try to acknowledge better the points that this reviewer has raised. Please note, as well, that we have updated the review to cover other relevant papers published since summer 2015, when this paper was submitted to the journal.

We chose not to attempt a fully systematic review since some formal systematic review procedures [5] are inappropriate for the type of problem and application of evidence considered here. We also considered it inappropriate to attempt meta-analysis or statistical or probabilistic assessments of the research evidence because: few studies that are relevant to this review follow strictly comparable methodologies; measures of change being used are quite diverse; some useful studies are qualitative not quantitative; the scope and scale of focus for the research reviewed is variable (including research at national,regional and local scales and various outcomes). We therefore adopted a 'structured' review approach, adapting the procedures required for formal systematic reviews.

The aims of the review were specified as shown above. We employed search and selection criteria designed to select research related to impacts of extreme events on factors relevant to health care system functioning, for which we were able to assess the rigor and quality of the research using quality criteria relevant to the type of (quantitative or qualitative) method used. We also selected papers reporting research related to the kinds of events and infrastructure relevant to health care functioning in the UK. We have not included papers reporting original research and reviews focussed on other country contexts, since, although there may be 'parallel' experiences in other countries, it is important to concentrate on the climate risks and built, organizational and social infrastructure in the UK national context [6]. Some more international studies were included if they report on internationally comparative work including the UK, or propose theoretical frameworks which seemed to have relevance to the UK setting.

The review procedure involved a first stage using an 'umbrella review' approach [5], drawing upon literature reviews previously prepared for projects supported by the LWEC Adaptation and Resilience to Climate Change programme (http://www.lwec.org.uk/activities/arcc) and by ESRC and EUFP7 (see acknowledgements). A number of key reviews and assessments produced recently for government agencies were also considered [3, 7-18]. In the second stage these reviews were supplemented by a 'structured' automated search for relevant literature

published 2010 – June 2017, on the online search engines Web of Science and PubMed, using the following terms: 'climate change', 'heat', 'cold', 'flood' in combination with 'health care', 'health services'. From the results of the automated search we made a manual selection of relevant articles meeting our criteria.*

Reviewer: This approach is a problem, in part, because the review of health effects is so ad hoc and slim. The first paragraph of that section about infrastructure should consider energy systems and the grid, more generally, as well as infrastructural factors that affect caring for those in-place when an extreme event occurs. Much of the healthcare necessary during such events will not happen in a hospital context

Response: This review is focussed on the health and social care system and we have concentrated on evidence for the UK. Some aspects of the impacts of extreme weather on population health, resulting in morbidity or mortality are not currently reflected in changes to health and social care system activity, so they are not discussed in this part of the review. Please note that we also wished to avoid overlap with other reviews to be included in this special issue. Where we found evidence relating to observed impacts on care systems outside hospitals we have included it, but the reviewer is correct that there is less reliable evidence for other parts of the health care system in in the UK.

Reviewer: Power is considered in the flood section and should also be considered in the heat section.

Response: We agree that for reviews of conditions in other countries, this would be appropriate, but we do explain (p 4) that for the UK this was not appropriate.

Reviewer: I suggest that the section "Findings regarding potential future impacts of extreme weather on health services demand due to mortality and morbidity in the UK" should come first before the more focused sections on specific kinds of events as a way to focus the paper.

Response: We considered this proposal, but we felt is more logical to present first the literature relating to observed impacts on services, then to discuss evidence about how health and care systems might be impacted in future, which is necessarily more speculative.

Reviewer: There are certain statements that need much greater substantiation. For example: "A number of sources argue that in addition to mitigation of climate change through more environmentally sustainable practices [83-85], strategies to adapt to climate change are important." This sentence does not sufficiently summarize an extremely large literature on adaptation. This is true of several other sentences in this paragraph.

Response: Thanks for picking this up. We have clarified this sentence, which now reads: 'environmentally sustainable practices in care systems', and reads as follows:

A number of sources argue that in addition to mitigation of climate change through more environmentally sustainable practices in care systems [89-91], strategies to adapt to climate change are important.

Reviewer: Another example is: “Few studies have assessed whether public awareness campaigns are effective for the most socially isolated and marginalized groups, who may be most vulnerable, but there is evidence that many people do not practice the public health advice they receive, e.g. older individuals do not always consider themselves vulnerable.” That can be referenced with a number of sources.
Response: This paragraph now includes references identified in our review which make these points:

Few studies have assessed whether public awareness campaigns are effective for the most socially isolated and marginalized groups, who may be most vulnerable, but there is evidence that many people do not practice the public health advice they receive, e.g. older individuals do not always consider themselves vulnerable [106, 113]. Extreme weather warnings can be confusing when issued from different sources and appearing to offer differing advice [112]. It is difficult to assess whether public awareness is improving over time, since many information programmes have only recently begun, extreme weather events tend to be rare, and the same populations may not be exposed repeatedly to similar events [112].

Reviewer: The most interesting part of the article starts in paragraph three of the section on improving infrastructure design. Here, it seems like the authors have thoroughly reviewed the literature. However, when the paper gets to the individual-level factors, it is very superficial and misses literature that has been produced in other countries but that could be applied to the UK context.
Response: We argue at the outset, with reference to literature on the significance of context, that it may not be appropriate to extend all of the international literature on individual level factors to the UK context. Please also see our response to the second reviewer, noting that in the last part of the paper (pages 9-10) we have selectively cited some of the international literature on approaches to adaptation and mitigation, which the reviewer may also have had in mind (including several examples published after we originally submitted this paper).

Reviewer: It seems that the section on risk identification is also referring to vulnerability, but does not reference the large literature that discusses related factors.
Response: We have added further reference to relevant work on national level vulnerability indicators developed in the UK.

Reviewer 2 Linda Varangu

Reviewer: Thanks to the authors for tackling this topic as it is of high importance for the health care sector around the world. In Canada we follow with great interest the leadership demonstrated by the UK and NHS on climate change resiliency and adaptation in the health care sector.

The paper as written is recommended for publication. A few comments with respect to the reported results are provided.

The broad objective of this research was to determine what information from peer-reviewed journals were available to help inform the effects of climate change, in particular extreme weather events of heatwaves, coldwaves and flooding, on the health and social services sector in the UK.

What this paper has shown is that according to the information presented in the peer-reviewed publications, there is still much to learn and to research when it comes to solid reporting on the impacts of climate change on the health care sector. While this is very important to know, it would also help if the authors could help us also understood some of the surrounding issues related to this observation and see how more of this reporting can be encouraged, especially in a more timely manner. We see in Canada, for example, some increase in reporting in the lay literature on extreme weather impacts on the health care sector particularly over the past several years, but this reporting is generally not accompanied by rigorous academic evaluations, nor is much of it get reported in peer-reviewed journals.

Response: This is a valid point. We have chosen to focus this structured review on publications that can be identified using data bases comprising peer reviewed journal. We have added the following sentence to acknowledge this point (p10).

We have noted arguments in the literature that it may be necessary to find ways to incorporate more qualitative ethnographic information describing individual experiences, or perhaps to be more responsive to lay reports. The growth in use of social media to track impacts of extreme events in real time may provide one mechanism for this.

Reviewer: The authors may want to consider the following observations on the challenges the health care sector representatives have in reporting on the impacts of climate change, which can include the following:

- Reporting by the health care sector on the impacts of climate change events and the effects on their operations and facilities, is generally targeting funders and community stakeholders.

- While we see some new reporting in the lay literature of the increased impacts of extreme weather, detailed assessments on these impacts on the health care sector are not easily available even in the lay literature. We know from media reports that impacts have occurred, such as temporary hospital closures due to power failures during extreme weather, but follow-up information is not easily available. This may be due to numerous competing health care sector priorities, lack of staff to undertake these more detailed reports, and engagement of staff who may not be well informed on the relationship of these events with the larger realm of climate change impacts.
- Situation analysis from impacts of extreme weather on the health care sector has generally tended to deal with lowest cost, short-term fixes.
- Reflections on the longer-term system changes that would elaborate on the potential of preparedness and adaptation measures that may enhance resilience are not common, as the health care sector has generally not taken this long-term assessment perspective. For primarily publically funded health care (which both the UK and Canadian health systems are), planning in the health care sector is associated with funding, and that is tied to the funding decision, and regulatory requirements set by the parties in power.
- Some health care sector members may be concerned that reporting on the effects of extreme weather on their facilities may be perceived as poor operations on their part, or that their facilities have not responded in ways that would attract future funders to their foundations. In such cases public reports would not be easily available.

Response:

These are all very interesting observations, warranting attention. We have tried to limit our discussion here to topics which are directly invoked by the literature reviewed, especially in view of comments from the other reviewer, who recommends a focussed review method. Also, the word limit for this article prevents us from elaborating on these points. However, we do include the following paragraph (updated with some references published since we submitted this paper to review) that now reads:

Local government policies in place are not always effectively implemented on the ground
[52, 54, 65, 128]. Sustainable care should be based on interdisciplinary research co-production, mutualism and localism [129, 130] and local knowledge needs to be integrated more effectively with environmental science on risks such as flooding [4, 131]. Research reports effective approaches using local case studies [54, 132, 133] [106] [134]. Similar emphasis on integrated planning comes from the international literature [71]. In Canada, Critical Systems Heuristics (CSH) was helpful in engaging

stakeholders, and addressing issues of power relations between collaborating partners [135] and recent research has compared and reviewed of a variety of toolkits now available [136] . International literature also emphasises stakeholder engagement and locally adapted frameworks to access critical social infrastructure involving connectedness, collaboration and adaptive response. While our review was not internationally comprehensive, we note examples of research in Canada [131] [137] the USA [138] [139] the UK [120] and Australia [140], with calls for coordinated action to; reduce heat exposure; improve access to cooling; adapt the built environment; enhance surveillance and early warning systems and public awareness communications [141]. Reviews also emphasise the need for a greater attention to ***equity and inclusivity****, drawing attention to the inequalities between socio-demographic groups in exposures to and impacts of extreme weather [142] and the increased care burdens that women are expected to undertake during disasters involving extreme weather events, that may be unsustainable [132]. Collective action at local level must pay attention to inclusive representation of subgroups within communities [143].*

Also, the conclusion now includes the following paragraph:

The recent literature reports on increasing use of new methods of tracking and studying impacts of extreme weather on health systems, including syndromic surveillance systems in the UK. We have noted arguments in the literature that it may be necessary to find ways to incorporate more qualitative ethnographic information describing individual experiences, or perhaps to be more responsive to lay reports. The growth in use of sources such as social media to track impacts of extreme events in real time provides one mechanism for this, which we may expect to see extended in countries like the UK.

We have also picked up in the conclusion on the very valid point about the impact of austerity programmes on action to improve adaptation, as follows:

Adaptation and preparedness measures may be hampered by pressures of terms of expenditure and competing demands on health care systems like that in the UK, which are mainly publicly funded. We have noted above some examples of econometric studies which have begun to explore the cost effectiveness of measures to make health and social care more resilient to extreme weather.

Reviewer: In order to plan for appropriate adaptation responses for the health care sector, we will need more information from the health care sector on what their impacts have been, and to have this information in a timely fashion, and assessed in a rigorous manner. Reporting could be facilitated by providing guidance to the health care sector on what information to report, such as through a standard reporting format, that will allow the further analysis by the academic community. This would require a greater partnership (and funding) for these types of analysis but could stimulate more publications in peer-reviewed journals, or more detailed

reports in the lay literature that may be acceptable for research reviews such as this one.

Response: This is a very good point. Again, we don't really have space, given the word limit to expand on this, but we do note examples from the UK of the growing literature on Syndromic Surveillance techniques, which are beginning, in the context of the UK National Health Service, to provide sufficiently comprehensive and systematically recorded data for this kind of assessment. We updated the review with some recently published examples of this approach. (eg see refs 44-46, 57, 133)

Also, in the last part of the paper (pages 9-10) we have selectively cited some of the international literature which the reviewer may have had in mind (including several examples published after we originally submitted this paper). This part now reads:

The international literature argues for ***more integrated planning and preparedness*** *for extreme weather, acknowledging the complex interdependencies involved [121], and the Sendai Framework for Disaster Risk Reduction underlines in an international context why this is important for health and wellbeing [122]. Commentators have underlined the need for interdisciplinary holistic strategies [123] to integrate robust, consistent scientific knowledge with local knowledge to improve resilience [70] [124], to emphasise sustainability and sustainable development [125] as a concern for health and social care systems, and to integrate human health and environmental impact analysis more effectively [126]. A growing emphasis on complexity theory and 'nexus' thinking [106, 127] provides frameworks that may help agencies and communities to develop strategies for preparedness for extreme weather that consider the networks of built, institutional and social infrastructures that are important for resilience.*

Local government policies in place are not always effectively implemented on the ground
[52, 54, 65, 128]. Sustainable care should be based on interdisciplinary research co-production, mutualism and localism [129, 130] and local knowledge needs to be integrated more effectively with environmental science on risks such as flooding [4, 131]. Research reports effective approaches using local case studies [54, 132, 133] [106] [134]. Similar emphasis on integrated planning comes from the international literature [71]. In Canada, Critical Systems Heuristics (CSH) was helpful in engaging stakeholders, and addressing issues of power relations between collaborating partners [135] and recent research has compared and reviewed of a variety of toolkits now available [136] . International literature also emphasises stakeholder engagement and locally adapted frameworks to access critical social infrastructure involving connectedness, collaboration and adaptive response. While our review was not internationally comprehensive, we note examples of research in Canada [131] [137] the USA [138] [139] the UK [120] and Australia [140], with calls for coordinated action to; reduce heat exposure; improve access to cooling; adapt the built environment;

enhance surveillance and early warning systems and public awareness communications [141]. Reviews also emphasise the need for a greater attention to ***equity and inclusivity****, drawing attention to the inequalities between socio-demographic groups in exposures to and impacts of extreme weather [142] and the increased care burdens that women are expected to undertake during disasters involving extreme weather events, that may be unsustainable [132]. Collective action at local level must pay attention to inclusive representation of subgroups within communities [143].*
